# Supplementary material for: Cryo-EM structure of hnRNPDL-2 fibrils, a functional amyloid associated with limb-girdle muscular dystrophy D3
Source: Nat Commun. 2023 Jan 16;14:239. doi: 10.1038/s41467-023-35854-0 (PMC9842712; doi:10.1038/s41467-023-35854-0)
Supplement: Supplementary file 1 — Supplementary Information [file 41467_2023_35854_MOESM1_ESM.pdf]

SUPPLEMENTARY INFORMATION

Cryo-EM structure of hnRNPDL-2 fibrils, a functional amyloid associated with limb-girdle muscular dystrophy D3

Javier Garcia-Pardo[a], Andrea Bartolomé-Nafría[a], Antonio Chaves-Sanjuan[bc], Marcos Gil-Garcia[a], Cristina Visentin[b,d], Martino Bolognesi[b,c], Stefano Ricagno[b,d] and Salvador Ventura[a,*]

[a]Institut de Biotecnologia i de Biomedicina (IBB) and Departament de Bioquímica i Biologia Molecular, Universitat Autònoma de Barcelona, 08193 Bellaterra, Barcelona, Spain.

[b]Dipartimento di Bioscienze, Università degli Studi di Milano, Milano 20133, Italy.

[c] CRC Fondazione Romeo e Enrica Invernizzi and NOLIMITS, Università degli Studi di Milano, Milano 20133, Italy.

[d]Institute of Molecular and Translational Cardiology, I.R.C.C.S. Policlinico San Donato, piazza Malan, 2, 20097, San Donato Milanese, Italy.

[*] To whom correspondence should be addressed: Salvador Ventura, Institut de Biotecnologia i de Biomedicina (IBB) and Departament de Bioquímica i Biologia Molecular, Universitat Autònoma de Barcelona, 08193 Bellaterra, Barcelona, Spain. Phone: +34-93-586 8956; Fax: +34-93-581 1264; E-mail: Salvador.Ventura@uab.cat.

TABLE OF CONTENT

SUPPLEMENTARY TABLES…………………………………………………………...3

Supplementary Table 1. Cryo-EM data collection and refinement statistics. 3

Supplementary Table 2. Solvent-accessible surface area (SASA) parameters calculated for the upper layers of hnRNPDL-2, hnRNPA2, hnRNPA1, hSAA1, Aß-42 and α-synuclein amyloid fibrils. 4

Supplementary Table 3. Solvent-accessible surface area (SASA) parameters calculated for the inner layers of hnRNPDL-2, hnRNPA2, hnRNPA1, hSAA1, Aß-42 and α-synuclein amyloid fibrils. 4

Supplementary Table 4. Solvation free energy gain per molecule (ΔG) and per residue (ΔG/residue) parameters calculated for hnRNPDL-2, hnRNPA2, hnRNPA1, α-synuclein, hSAA1 and Aß-42 amyloid fibrils. 5

Supplementary Table 5. Calculated Gibbs free energy values upon mutations on hnRNPDL-2, hnRNPA1 and hnRNPA2 amyloid fibrils. 5

SUPPLEMENTARY FIGURES…………………………………………………….............6

Supplementary Fig. 1. Sequence alignment of hnRNPDL isoforms. 6

Supplementary Fig. 2. Characterization of HeLa hnRNPDL knockout (KO) cell line. 7

Supplementary Fig. 3. Nuclear localization of GFP-tagged hnRNPDL isoforms in HeLa cells 8

Supplementary Fig. 4. hnRNPDL isoforms amyloid formation. 9

Supplementary Fig. 5. Effect of NaCl on hnRNPDL isoforms amyloid formation. 10

Supplementary Fig. 7. Effect of NaCl and temperature on hnRNPDL fibril stability. 12

Supplementary Fig. 8. hnRNPDL-2 forms non-toxic amyloid fibrils 13

Supplementary Fig. 9. Detergent-soluble and insoluble fractions of cellular endogenous hnRNPDL-2 in HeLa cells. 14

Supplementary Fig. 11. Resolution estimation of hnRNPDL-2 amyloid filaments. 16

Supplementary Fig. 12. Relevant interactions in the hnRNPDL-2 protofilament interface. .. 17

Supplementary Fig. 13. hnRNPDL-2 fibrils display a highly polar surface interface. 18

Supplementary Fig. 14. Stability of hnRNPDL-2 in comparison with other functional and pathological amyloid fibrils. 19

Supplementary Fig. 15. Comparison of cryo-EM structures of amyloid fibrils from RNA/DNA binding proteins. 20

Supplementary Fig. 16. Predicted energy for fibrillation of six-residue windows of hnRNPDL-2 and hnRNPA2 disease-associated mutants. 21

Supplementary Fig. 18. Structure of the RNA/DNA-binding domains from hnRNPDL-2. ... 23

Supplementary Fig. 19. RNA (F-GACUAGC) binding to soluble hnRNPDL-2. 24

Supplementary Fig. 20. Location of amyloid cores and conserved exons in hnRNPDL, hnRNPA1 and hnRNPA2. 25

SUPPLEMENTARY REFERENCES 26

SUPPLEMENTARY TABLES

Supplementary Table 1. Cryo-EM data collection and refinement statistics.

**Name**	**hnRNPDL-2 amyloid fibril**
**PDB ID**<br>**EMDB ID**<br>**EMPIAR**	**7ZIR**<br>**EMDB-14738**<br>**EMPIAR-11064**
**Data collection**	
Magnification	×120000
Pixel size (Å)	0.889
Defocus range (μm)	-1.0 to -2.5
Voltage (kV)	200
Camera	Falcon 3EC
Microscope	Talos Arctica
Number of frames	40
Total dose ($e^-/Å^2$)	40
**Reconstruction**	
Micrographs	1114
Manually picked fibrils	10450
Box size (pixel)	380
Inter-box distance (Å)	33.25
Segments extracted	158493
Segments after Class2D	158493
Segments after Class3D	54490
Resolution (Å)	2.5
Map sharpening B-factor ($Å^2$)	-46.0
Helical rise (Å)	4.82
Helical twist (°)	- 4.86
**Atomic model***	
Non-hydrogen atoms	2120
Protein residues	255
Water molecules	50
r.m.s.d. Bond length	0.001 (0)
r.m.s.d. Bond angle	0.300 (0)
All-atom clash score	2.48
Rotamers outliers	0
Ramachandran outliers (%)	0
Ramachandran allowed (%)	2.04
Ramachandran favored (%)	97.96

*Note that the atomic model accounts for five layers, one chain each, of the hnRNPDL-2 amyloid core.

Supplementary Table 2. Solvent-accessible surface area (SASA) parameters calculated for the upper layers of hnRNPDL-2, hnRNPA2, hnRNPA1, hSAA1, Aß-42 and α-synuclein amyloid fibrils.

	**Total Upper Surface (Å^2)**	**Buried Upper Surface (Å^2)**	**Exposed Upper Surface (Å^2)**	**Exposed Polar Surface (%)**	**Polar Buried Residues (%)**
**hnRNPDL-2**	5789	2330	3458	48	64
**hnRNPA1**	4721	2480	2241	42	47
**hnRNPA2**	5761	2382	3379	42	52
**hSAA1**	6482	3078	3404	38	47
**α-synuclein**	6168	2521	3648	36	42
**Aβ-42**	3644	1706	1938	32	31

Note that proteins are ordered according to decreasing Exposed Polar Surface (%) values. Exposed Polar (%) values were calculated using GetArea[1]. Surfaces values and polar buried residues (%) were calculated with PDBePISA[2]. PDB accession codes used for calculations are: hnRNPDL-2 (PDB 7ZIR, residues 345-395 according to hnRNPDL-1 numbering), hnRNPA2 (PDB 6WQK, residues 263-319), hnRNPA1 (PDB 7BX7, residues 251-295), hSAA1 (PDB 6MST, residues 2-55), Aβ-42 (PDB 5KK3, residues 11-42) and α-synuclein (PDB 6OSJ, residues 37-97).

Supplementary Table 3. Solvent-accessible surface area (SASA) parameters calculated for the inner layers of hnRNPDL-2, hnRNPA2, hnRNPA1, hSAA1, A□-42 and α-synuclein amyloid fibrils.

	**Total Inner Surface (Å^2)**	**Buried Inner Surface (Å^2)**	**Exposed Inner Surface (Å^2)**	**Exposed Polar Surface (%)**	**Polar Buried Residues (%)**
**hnRNPDL-2**	5761	4467	1294	57	67
**hnRNPA2**	5758	4518	1240	47	53
**hnRNPA1**	4721	4117	604	44	46
**hSAA1**	6477	5474	1003	43	46
**Aβ-42**	3589	2868	720	40	31
**α-synuclein**	6170	4741	1429	37	42

Note that proteins are ordered according to decreasing Exposed Polar Surface (%) values. Exposed Polar (%) values were calculated using GetArea[1]. Surfaces values and polar buried residues (%) were calculated with PDBePISA[2]. PDB accession codes used for calculations are: hnRNPDL-2 (PDB 7ZIR, residues 345-395 according to hnRNPDL-1 numbering), hnRNPA2 (PDB 6WQK, residues 263-319), hnRNPA1 (PDB 7BX7, residues 251-295), hSAA1 (PDB 6MST, residues 2-55), Aβ-42 (PDB 5KK3, residues 11-42) and α-synuclein (PDB 6OSJ, residues 37-97).

Supplementary Table 4. Solvation free energy gain per molecule (ΔG) and per residue (ΔG/residue) parameters calculated for hnRNPDL-2, hnRNPA2, hnRNPA1, *α*-synuclein, hSAA1 and Aß-42 amyloid fibrils.

	PDB accession code	ΔG (kcal/mol)	ΔG/residue (kcal/mol)
**hnRNPA1**	7BX7	-15.3	-0.34
**hnRNPA2**	6WQK	-20.7	-0.36
**hnRNPDL-2**	7ZIR	-21.7	-0.43
α*-synuclein	6XYP	-49.3	-0.61
α*-synuclein	6XYQ	-50.0	-0.62
α*-synuclein*	7NCA	-38.4	-0.63
α*-synuclein*	7NCH	-43.2	-0.64
**hsAA1***	6MST	-35.1	-0.65
α*-synuclein*	7NCI	-44.4	-0.65
α*-synuclein*	7NCG	-40.0	-0.66
α*-synuclein	6XYO	-53.3	-0.66
***α*-synuclein**	6OSJ	-40.2	-0.66
α*-synuclein*	7NCK	-45.5	-0.71
α*-synuclein*	7NCJ	-43.4	-0.71
**Aβ-42***	7Q4B	-26.5	-0.78
**Aβ-42**	5KK3	-21.9	-0.78
**Aβ-42***	7Q4M	-26.5	-0.85

Note that proteins are ordered according to increasing ΔG/residue values. ΔG values were calculated with PDBePISA[2]. *Fibrils obtained *ex vivo*. **Fibrils obtained upon seeding with *ex vivo* filaments.

Supplementary Table 5. Calculated Gibbs free energy values upon mutations on hnRNPDL-2, hnRNPA1 and hnRNPA2 amyloid fibrils.

Protein	Mutation	ΔΔG (kcal/mol)
**hnRNPDL-2**	D259H	2.50
	D259N	-5.54
**hnRNPA1**	D262N	-15.60
	D262V	-29.84
**hnRNPA2**	D290V	-9.33

SUPPLEMENTARY FIGURES

Supplementary Fig. 1

```
hnRNPDL-1    1- MEVPPRLSHVPPPLFPSAPATLASRSLSHWRPRPPRQLAPLLPSLAPSSARQGARRAQRH -60
hnRNPDL-2       ------------------------------------------------------------
hnRNPDL-3       ------------------------------------------------------------

hnRNPDL-1   61- VTAQQPSRLAGGAAIKGGRRRRPDLFRRHFKSSSIQRSAAAAAATRTARQHPPADSSVTM -120
hnRNPDL-2    1- -----------------------------------------------------------M -1
hnRNPDL-3    1- -----------------------------------------------------------M -1

hnRNPDL-1  121- EDMNEYSNIEEFAEGSKINASKNQQDDGKMFIGGLSWDTSKKDLTEYLSRFGEVVDCTIK -180
hnRNPDL-2    2- EDMNEYSNIEEFAEGSKINASKNQQDDGKMFIGGLSWDTSKKDLTEYLSRFGEVVDCTIK -61
hnRNPDL-3    2- EDMNEYSNIEEFAEGSKINASKNQQDDGKMFIGGLSWDTSKKDLTEYLSRFGEVVDCTIK -61

hnRNPDL-1  181- TDPVTGRSRGFGFVLFKDAASVDKVLELKEHKLDGKLIDPKRAKALKGKEPPKKVFVGGL -240
hnRNPDL-2   62- TDPVTGRSRGFGFVLFKDAASVDKVLELKEHKLDGKLIDPKRAKALKGKEPPKKVFVGGL -121
hnRNPDL-3   62- TDPVTGRSRGFGFVLFKDAASVDKVLELKEHKLDGKLIDPKRAKALKGKEPPKKVFVGGL -121

hnRNPDL-1  241- SPDTSEEQIKEYFGAFGEIENIELPMDTKTNERRGFCFITYTDEEPVKKLLESRYHQIGS -300
hnRNPDL-2  122- SPDTSEEQIKEYFGAFGEIENIELPMDTKTNERRGFCFITYTDEEPVKKLLESRYHQIGS -181
hnRNPDL-3  122- SPDTSEEQIKEYFGAFGEIENIELPMDTKTNERRGFCFITYTDEEPVKKLLESRYHQIGS -181

hnRNPDL-1  301- GKCEIKVAQPKEVYRQQQQQQKGGRGAAAGGRGGTRGRGRGQGQNWNQGFNNYYDQGYGN -360
hnRNPDL-2  182- GKCEIKVAQPKEVYRQQQQQQKGGRGAAAGGRGGTRGRGRGQGQNWNQGFNNYYDQGYGN -241
hnRNPDL-3  182- GKCEIKVAQPKEVYRQQQQQQKGGRGAAAGGRGGTRGRGRGQ------------------ -223
                                                                 NLS
hnRNPDL-1  361- YNSAYGGDQNYSGYGGYDYTGYNYGNYGYGQGYADYSGQQSTYGKASRGGGNHQNNYQPY -420
hnRNPDL-2  242- YNSAYGGDQNYSGYGGYDYTGYNYGNYGYGQGYADYSGQQSTYGKASRGGGNHQNNYQPY -301
hnRNPDL-3       ----------------------------------------QSTYGKASRGGGNHQNNYQPY -244
```

Supplementary Fig. 1. Sequence alignment of hnRNPDL isoforms. The amino acid sequences of hnRNPDL from three different majoritarian isoforms (isoform 1, hnRNPDL-1; isoform 2, hnRNPDL-2 and isoform 3, hnRNPDL-3) were aligned with Clustal Omega. Uniprot accession numbers are: hnRNPDL-1 (O14979-1), hnRNPDL-2 (O14979-2) and hnRNPDL-3 (O14979-3). The residues comprising the N-terminal Arg-rich LCD and RRMs are shown in orange and pink, respectively. The amino acids comprising the Gly/Tyr-rich amyloid exon 6 are depicted in blue. The Asp residue in position 259 (D259), which is mutated in LGMD D3 patients, is shown in red. The C-terminal amino acids that encode for the hnRNPDL nuclear localization signal (NLS) are indicated.

Supplementary Fig. 2

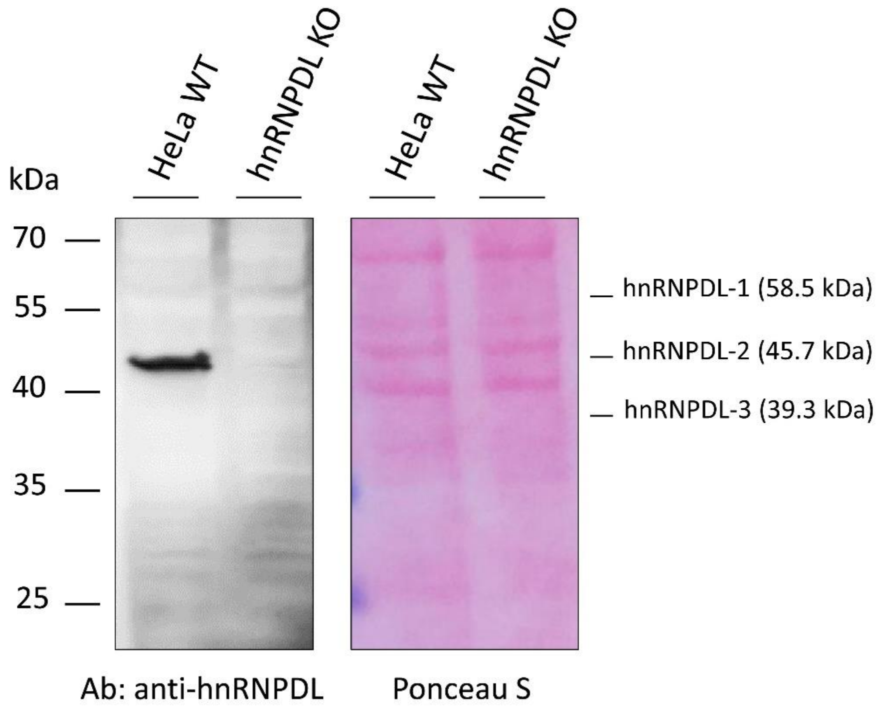

Supplementary Fig. 2. Characterization of HeLa hnRNPDL knockout (KO) cell line. Left panel: Western blot analysis of hnRNPDL expression levels in wild-type HeLa cells (WT) in comparison with hnRNPDL knockout HeLa cells (left panel). The presence of the protein in the soluble fraction was detected using a specific anti-hnRNPDL antibody. Right panel: Ponceau S staining of the same blot as loading control. This experiment has been performed three times with identical results.

Supplementary Fig. 3

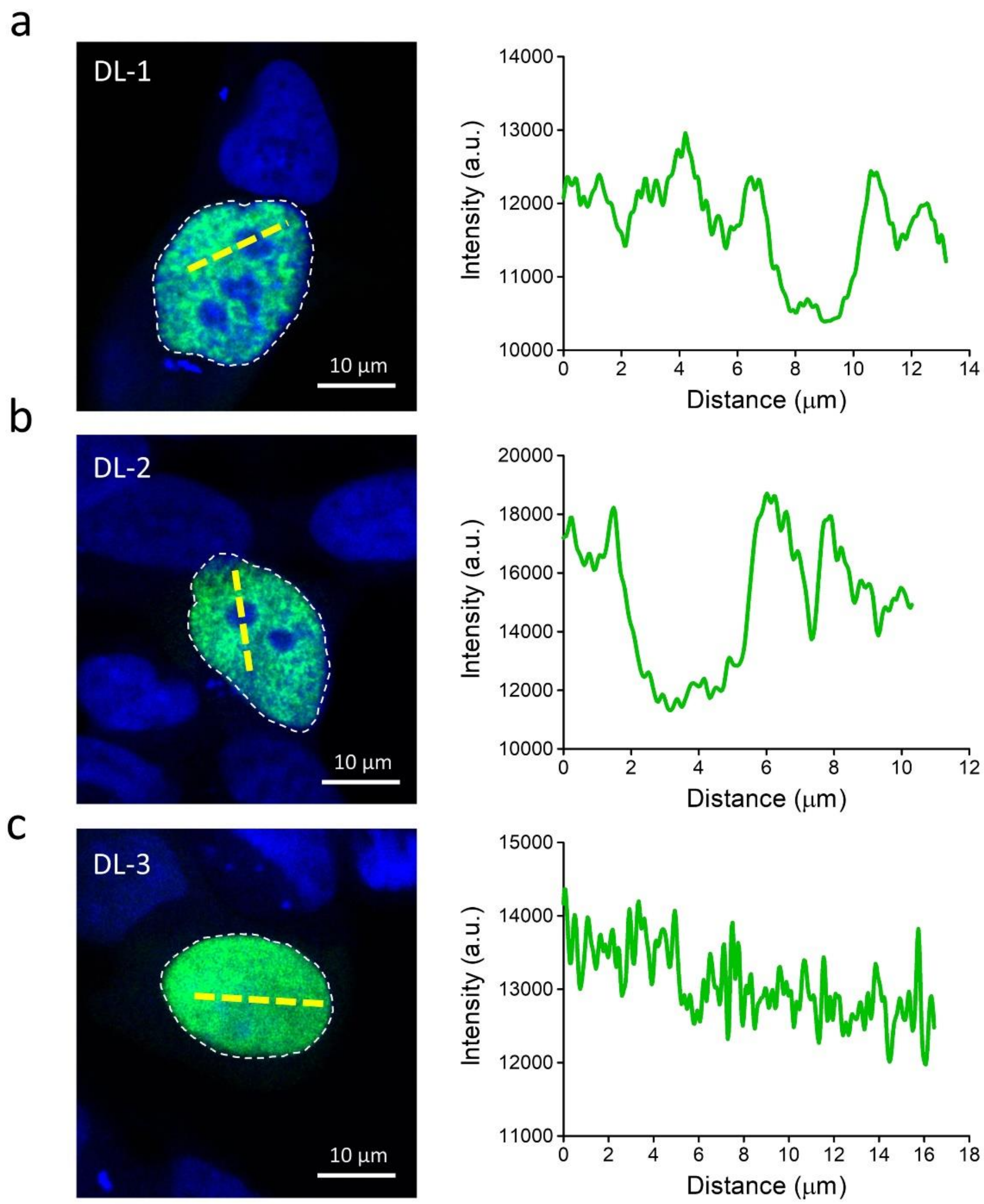

Supplementary Fig. 3. Nuclear localization of GFP-tagged hnRNPDL isoforms in HeLa cells. Left panels: Confocal images of HeLa hnRNPDL KO cells transiently transfected with the three hnRNPDL isoforms (**a**) hnRNPDL-1 (DL-1), (**b**) hnRNPDL-2 (DL-2), and (**c**) hnRNPDL-3 (DL-3). The images show the nuclear distribution of the different GFP-tagged proteins (green). Nuclear DNA was stained with Hoechst (blue). The nucleus contour has been highlighted with a white dashed line. Right panels: Intensity profile analyses of the regions of interest (ROI) shown in (**a-c**) as yellow dashed lines. These results are representative from three independent experiments; a.u. indicates arbitrary units.

Supplementary Fig. 4

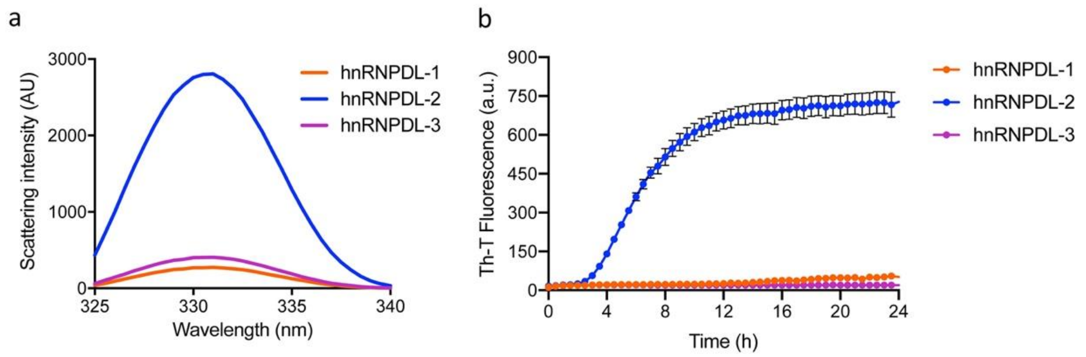

Supplementary Fig. 4. hnRNPDL isoforms amyloid formation. a Light scattering analysis of hnRNPDL-1 (orange line), hnRNPDL-2 (blue line) and hnRNPDL-3 (purple line) aggregates after incubation at 37 °C under shaking at 600 rpm for 48 h; AU indicates absorbance units. **b** Aggregation kinetics of hnRNPDL-1 (orange line), hnRNPDL-2 (blue line) and hnRNPDL-3 (purple line) at 25 µM. Aggregation is shown as Th-T binding over time and samples were incubated at 37 °C and under constant shaking in 96 well plates; a.u. indicates arbitrary units. In (**b**), data is shown as mean ± SEM of (n = 3 independent experiments).

Supplementary Fig. 5

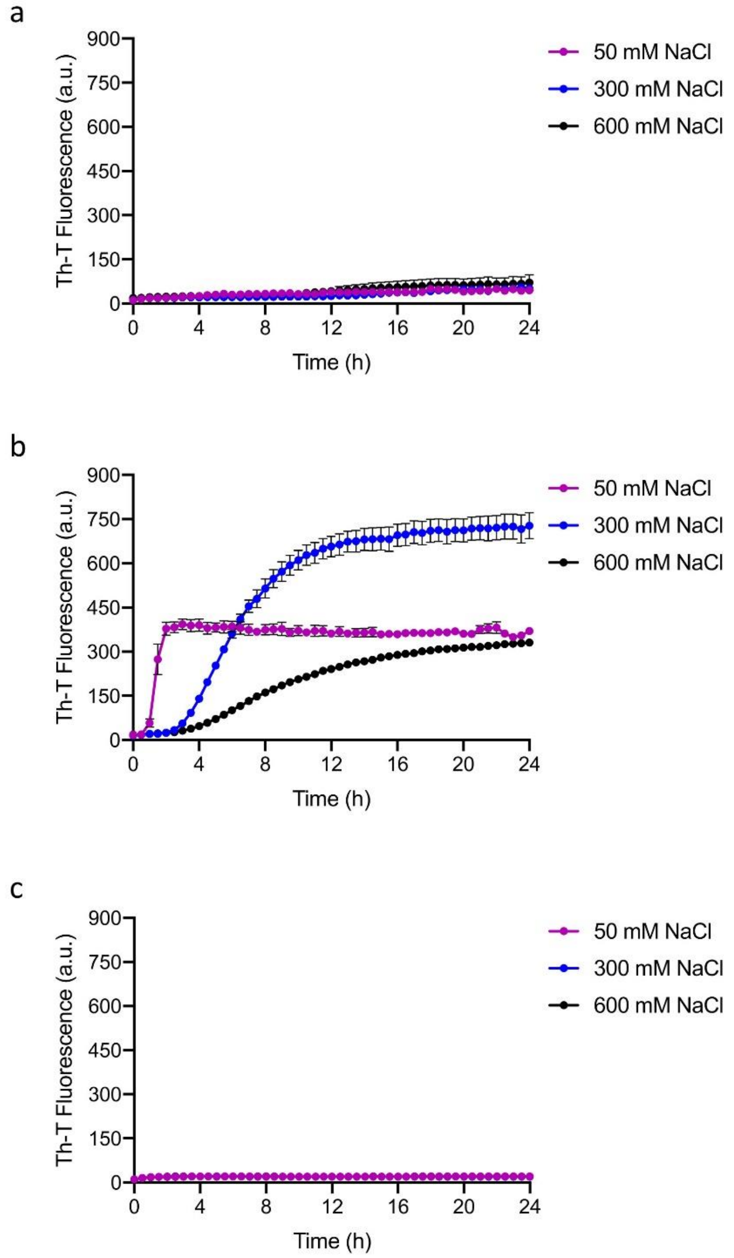

Supplementary Fig. 5. Effect of NaCl on hnRNPDL isoforms amyloid formation. a-c Aggregation kinetics of soluble (**a**) hnRNPDL-1, (**b**) hnRNPDL-2 and (**c**) hnRNPDL-3 determined at three different NaCl concentrations. Aggregation is shown as Th-T binding over time. Samples were incubated at 37 °C and under constant shaking in 96 well plates as described. In (**a-c**), data is shown as mean ± SEM (n = 3 independent experiments); a.u. indicates arbitrary units.

Supplementary Fig. 6

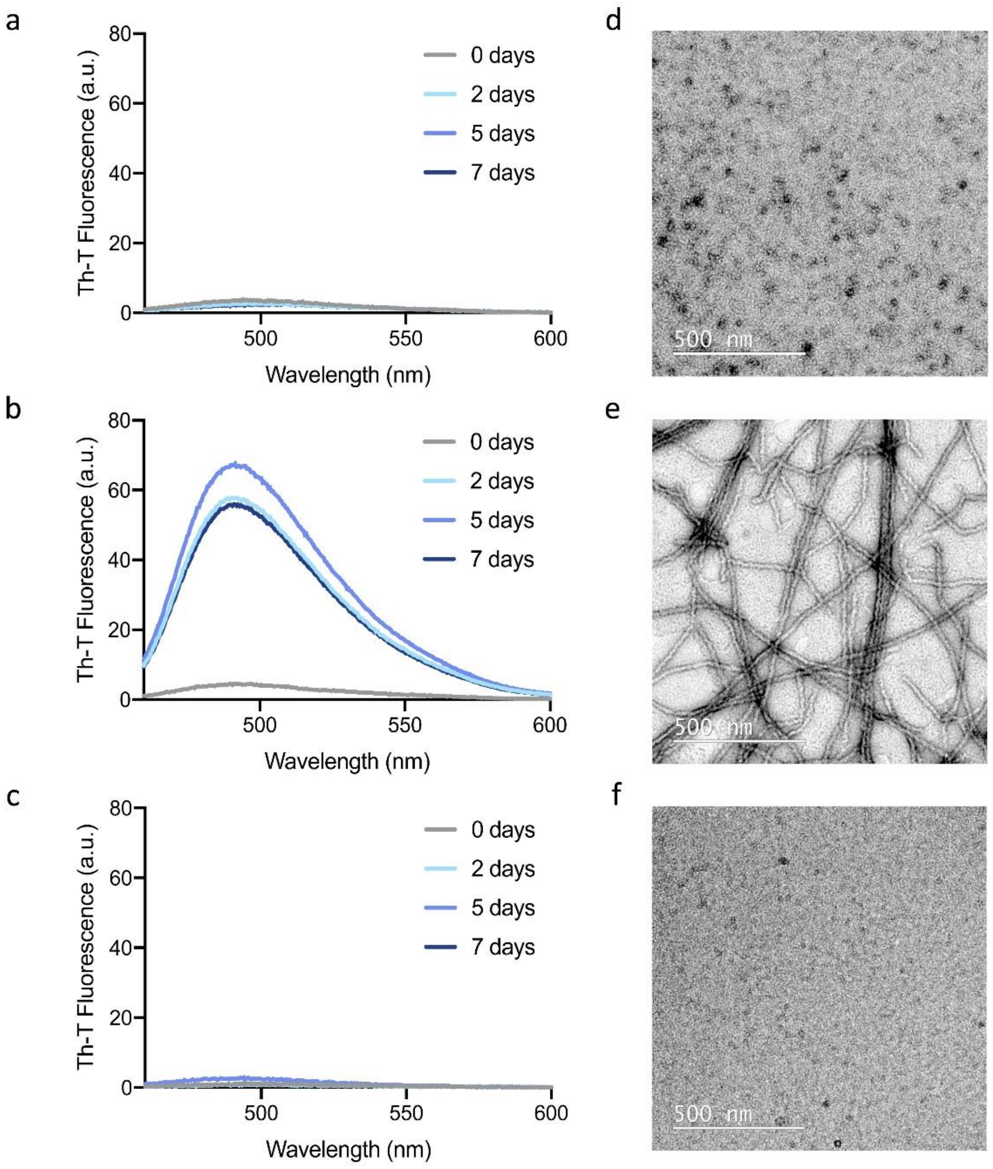

Supplementary Fig. 6. Effect of the incubation time on hnRNPDL-1, hnRNPDL-2 and hnRNPDL-3 aggregation. a-c Th-T binding to (**a**) hnRNPDL-1, (**b**) hnRNPDL-2 and (**c**) hnRNPDL-3 after incubation of the samples at 37 °C, pH 7.5 and 300 mM NaCl for 0, 2, 5 and 7 days of incubation. **d-f** Representative TEM micrographs of (**d**) hnRNPDL-1, (**e**) hnRNPDL-2 and (**f**) hnRNPDL-3 after 7 days incubation under the specified conditions. Similar results were obtained from three different aggregation experiments performed with samples from different protein purifications; a.u. indicates arbitrary units.

Supplementary Fig. 7

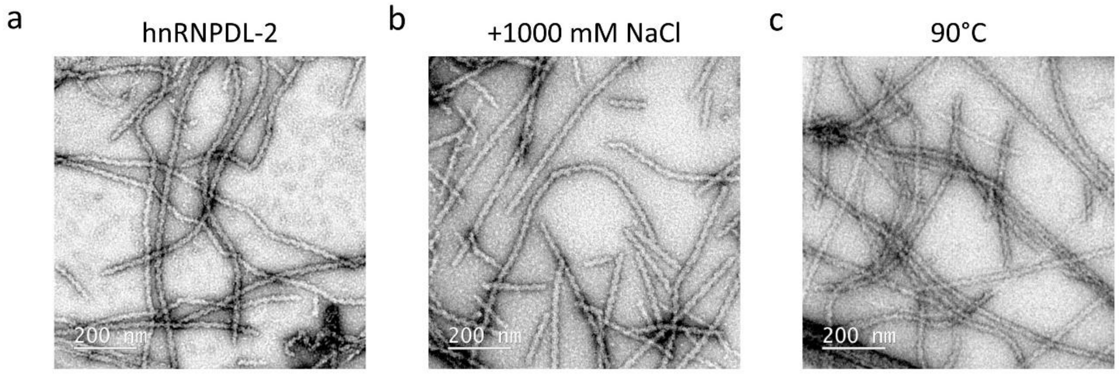

Supplementary Fig. 7. Effect of NaCl and temperature on hnRNPDL fibril stability. a-c Representative negative-staining TEM micrographs of pre-formed hnRNPDL-2 amyloid fibrils after 48 h in the (**a**) absence or (**b**) presence of 1000 mM NaCl, and (**c**) after 2h at 90 °C. Similar results were obtained from two different aggregation experiments performed with samples from different protein purifications.

Supplementary Fig. 8

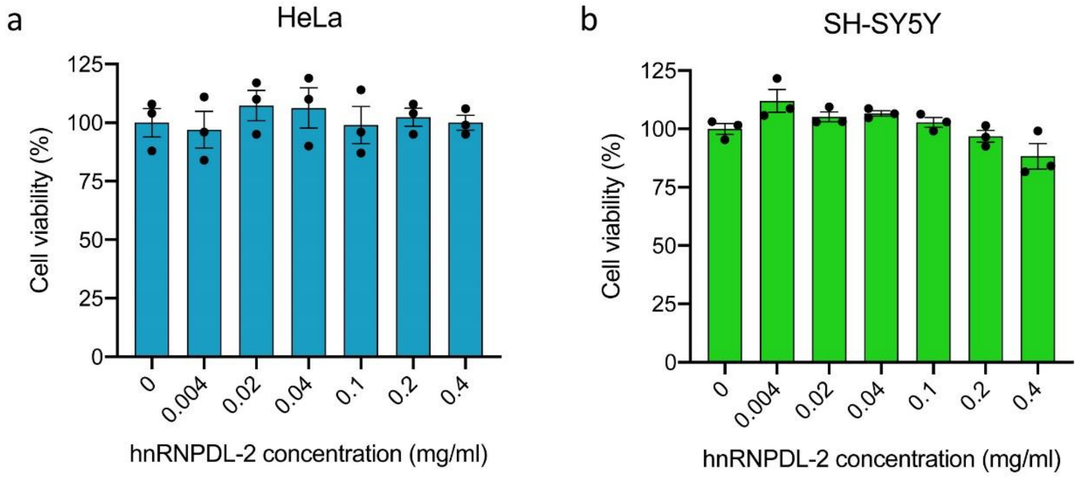

Supplementary Fig. 8. hnRNPDL-2 forms non-toxic amyloid fibrils. a-b Effect of hnRNPDL-2 amyloid fibrils on the viability of HeLa (**a**) or human neuroblastoma SH-SY5Y (**b**) cells after incubation with the pre-formed filaments for 48 h. Data is shown as mean ± SEM (n = 3 independent experiments).

Supplementary Fig. 9

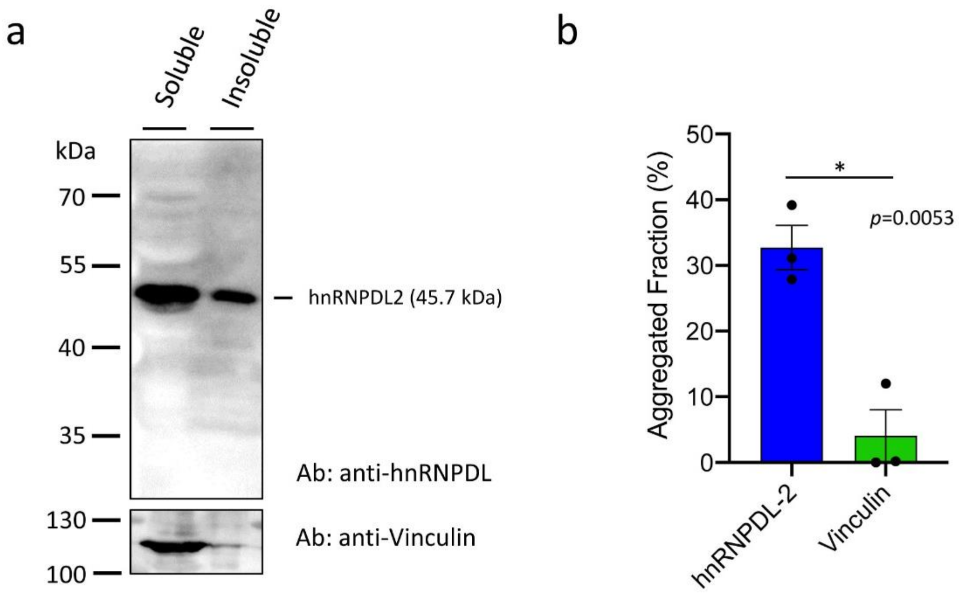

Supplementary Fig. 9. Detergent-soluble and insoluble fractions of cellular endogenous hnRNPDL-2 in HeLa cells. a Western blot analysis of the soluble and insoluble fraction of HeLa cells lysed with mPER Mammalian Protein Extraction Reagent. The presence of hnRNPDL-2 in soluble and insoluble fractions was detected by immunoblotting with anti-hnRNPDL antibody. The presence of vinculin in the different fractions was determined as control protein. These results are representative from three independent experiments. **b** Relative Levels (%) of hnRNPDL-2 or vinculin present in the aggregated fraction (detergent-insoluble fraction) of HeLa cells after cell lysis. In (**b**), data is shown as mean ± SEM (n = 3 independent experiments). Differences between means were analyzed by an unpaired two-tailed *t*-test (p=0.0053). *indicates $p < 0.05$.

Supplementary Fig. 10

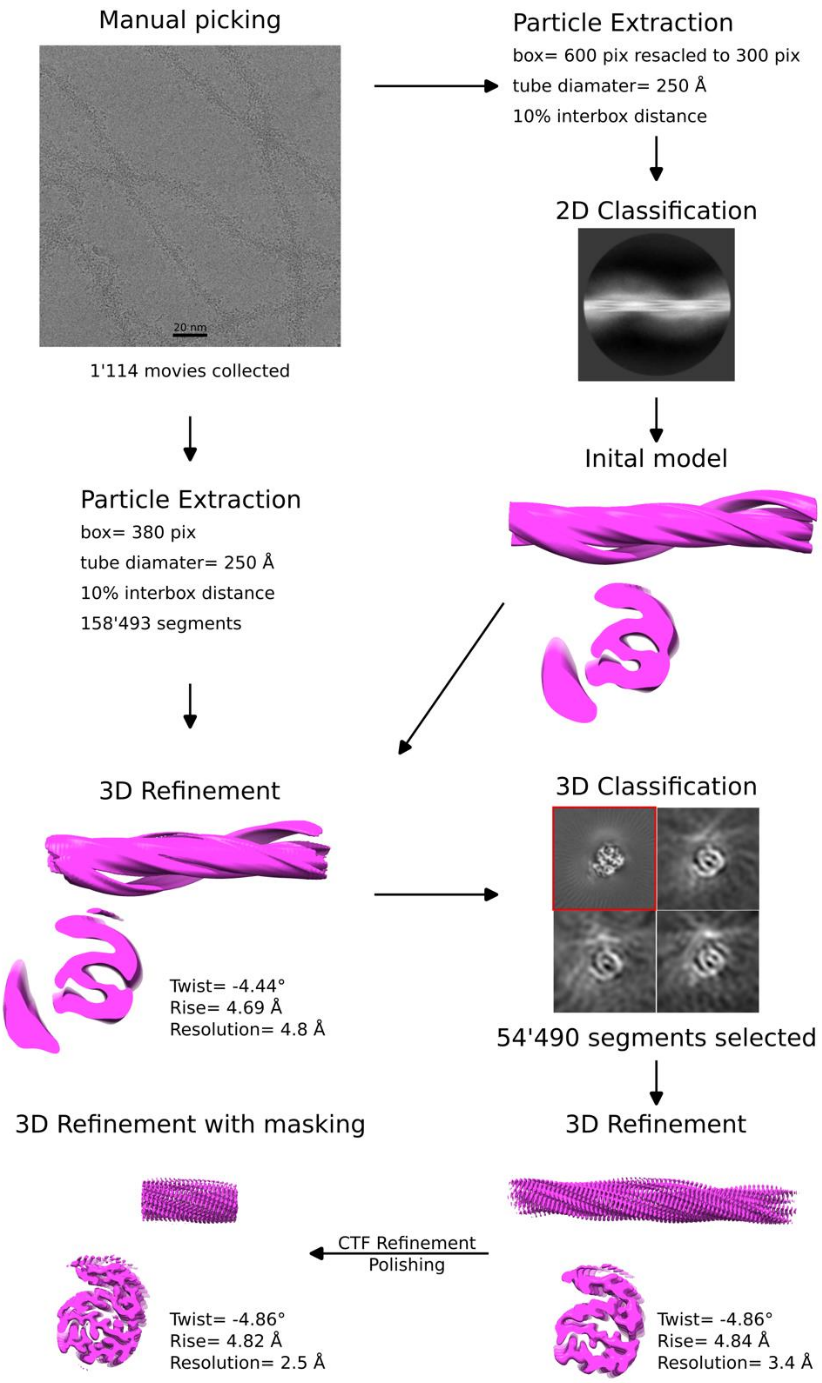

Supplementary Fig. 10. Cryo-EM data processing workflow. The diagram shows representative steps in the image processing workflow, connected by arrows. Key settings and result values are specified for each step. One micrograph and a 2D average from the manual picking and 2D classification stages are shown. The four classes from the 3D classification step are shown as slices with the selected one marked in red. Two views of the initial and of the refinement reconstructions are shown. The full protocol is detailed in the Methods section.

Supplementary Fig. 11

a

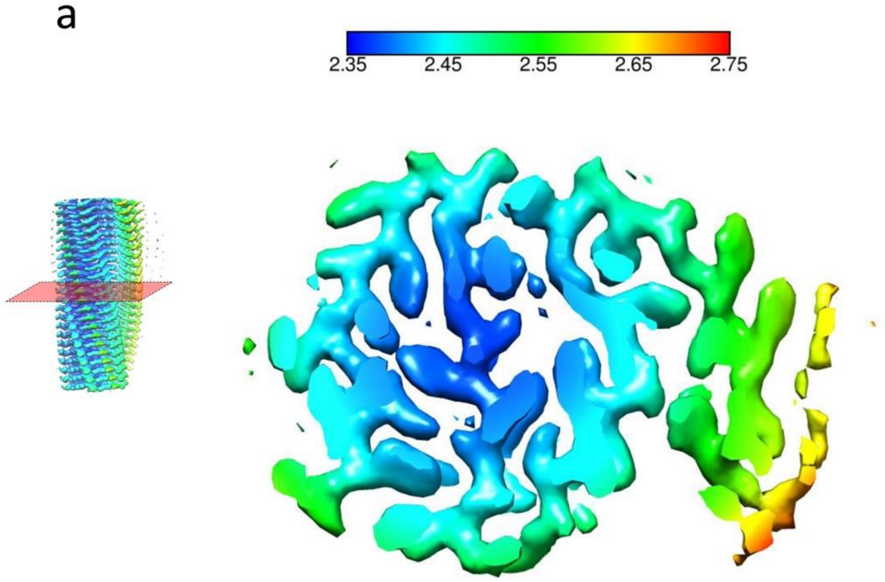

b

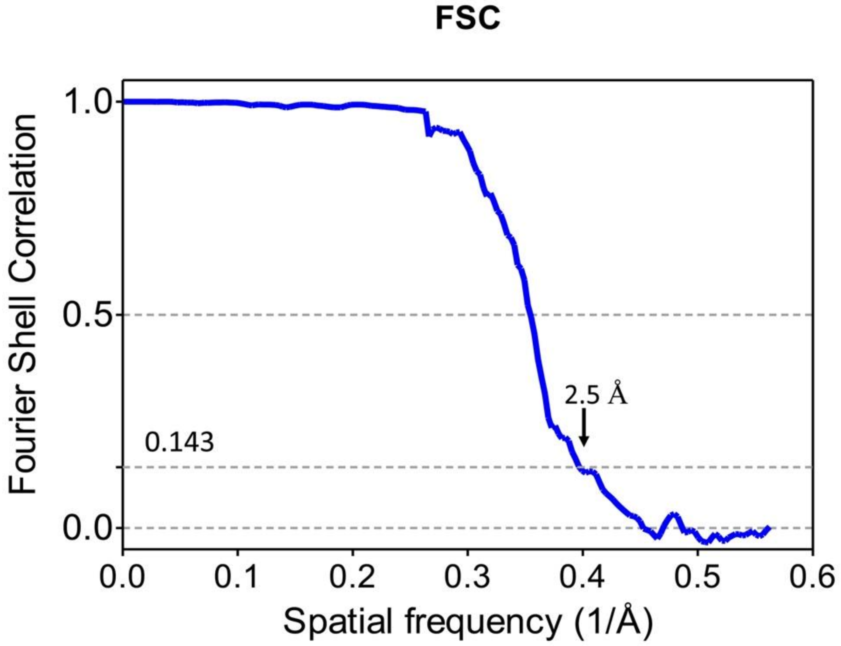

Supplementary Fig. 11. Resolution estimation of hnRNPDL-2 amyloid filaments. a Local resolution map of the hnRNPDL-2 filament reconstruction; local resolution levels (Å) are color coded on top. **b** Fourier Shell Correlation curve (FSC) of hnRNPDL-2 amyloid filaments. The overall resolution of the best reconstruction is 2.5 Å (FSC=0.143).

Supplementary Fig. 12

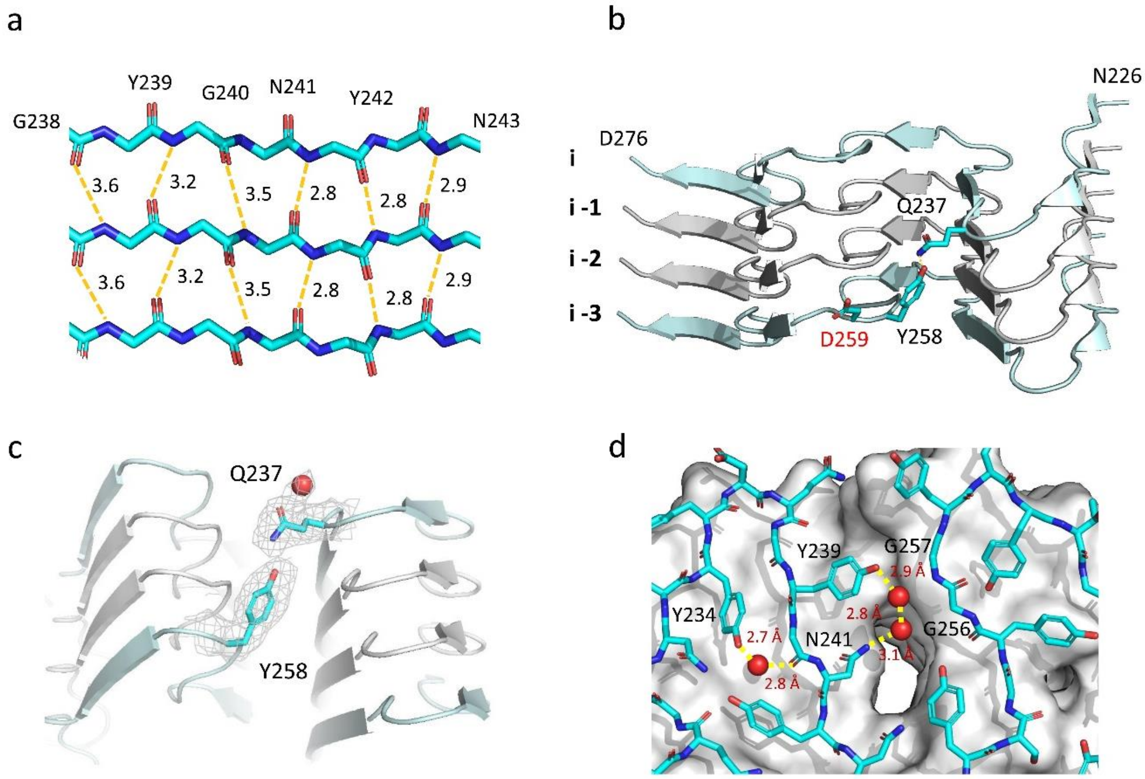

Supplementary Fig. 12. Relevant interactions in the hnRNPDL-2 protofilament interface. a Close-up view of the inter-molecular hydrogen-bonding network holding consecutive parallel β-strands between residues G238 and N243. **b** Side view of four layers of the amyloid core of hnRNPDL-2, depicting the interaction between the side chains of Q237 and Y258. The side chain of D259, which is mutated in LGMD D3, is also shown as sticks. **c** Close up view of the residues involved in the interaction between the layers i and i -3. The side chains of Q237 and Y258 are shown as sticks colored in cyan over the cryo-EM map sown as a grey mesh. **d** Close-up view of the central water channel found in the hnRNPDL-2 amyloid core. Relevant hydrogen bonds stablished between water molecules in the channel and the adjacent residues from the first layer are depicted (yellow dashed lines). The corresponding distances are indicated in Å.

Supplementary Fig. 13

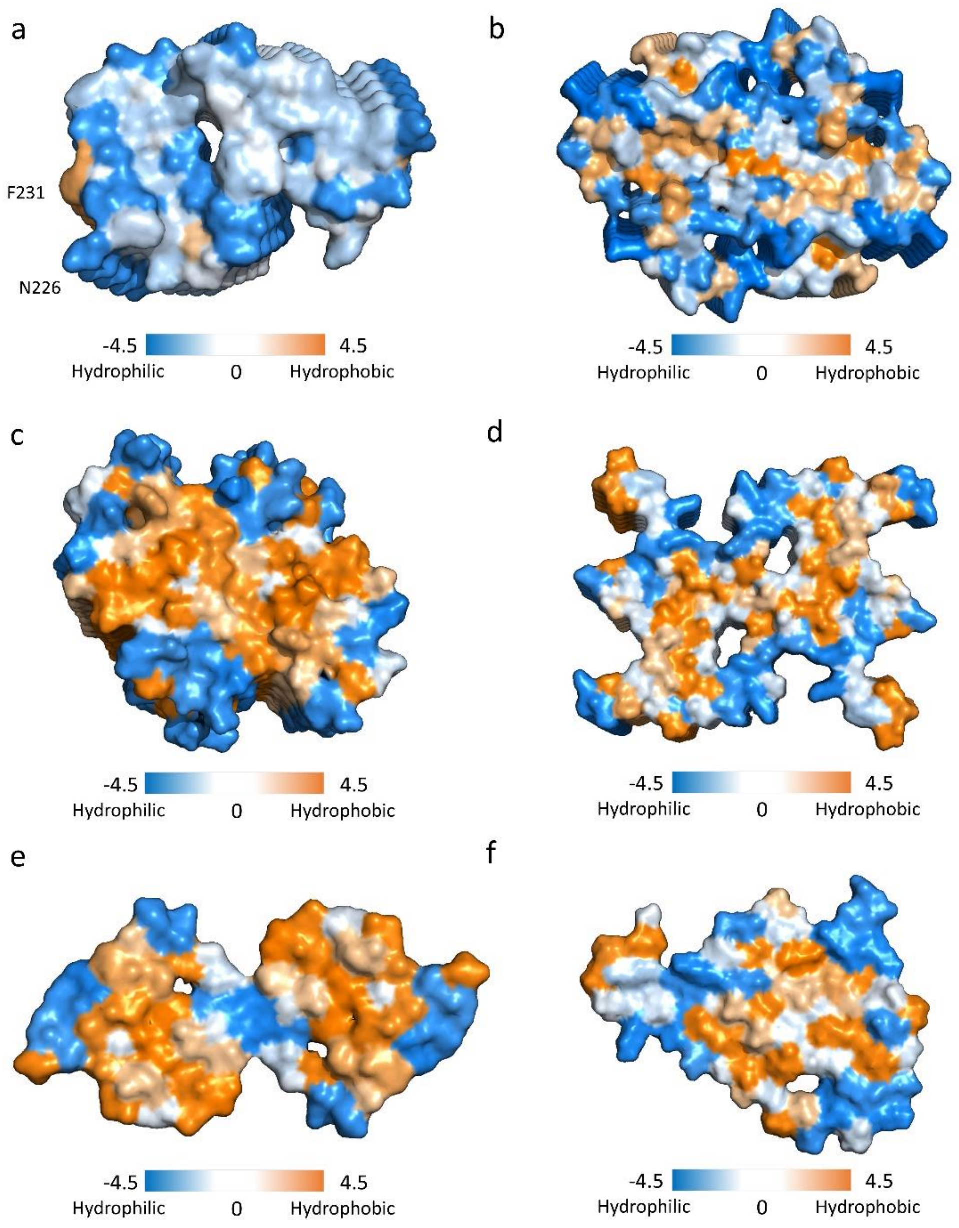

Supplementary Fig. 13. hnRNPDL-2 fibrils display a highly polar surface interface. Surface representation of (**a**) hnRNPDL-2 (PDB 7ZIR), (**b**) human serum amyloid A (PDB 6MST), (**c**) Amyloidβ (Aβ)-42 (PDB 5KK3), (**d**) α-synuclein (PDB 6OSJ), (**e**) type 2 Aβ-42 (PDB 7Q4M) and (**f**) type 3 α-synuclein (PDB 7NCK) fibrils cross-sections colored with the hydrophobicity levels of each residue. Note that (**a**), (**c**) and (**d**) are amyloid fibrils obtained *in vitro*, while (**b**) and (**e**) constitute two examples of pathological amyloids obtained *ex vivo* from human tissues and (**f**) is an amyloid fibril seeded by ex vivo filaments. hnRNDPL-2 has a unique strong hydrophobic spot visible in the core of the fibril. This region corresponds to the side chain of F231, which is located near the N-terminus. The hydrophobicity levels were assigned to each residue according to the Kyte-Doolittle scale[3].

Supplementary Fig. 14

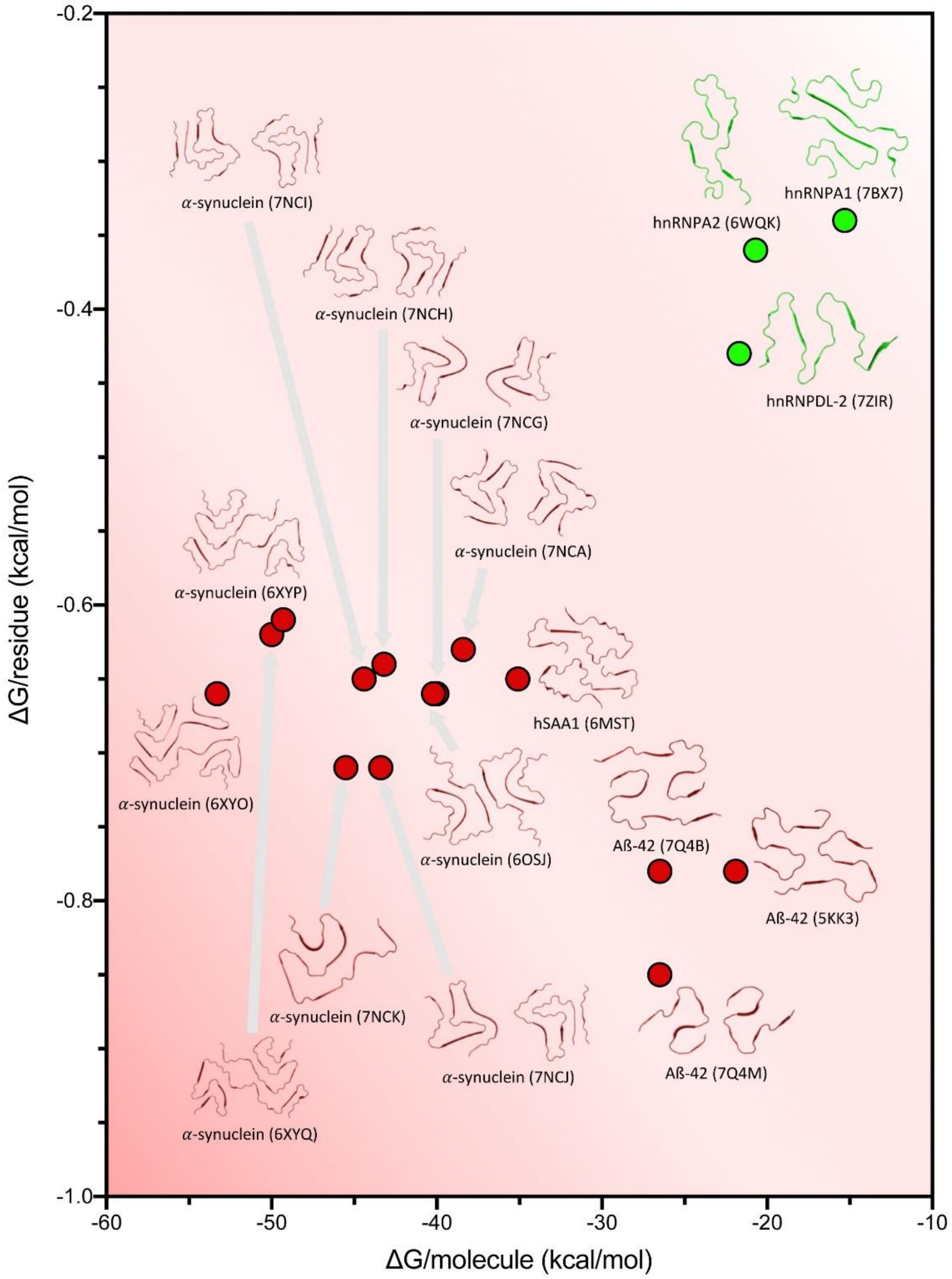

Supplementary Fig. 14. Stability of hnRNPDL-2 in comparison with other functional and pathological amyloid fibrils. Represented with fibril structures in two dimensions: solvation free energy gain (ΔG in Kcal/mol) per molecule (horizontal axis) and per residue (vertical axis). ΔG values were calculated with PDBePISA2. Amyloid fibril structures that are non-pathogenic and less stable are colored in green, whereas pathogenic and more stable amyloid filaments are colored in red. Note that the corresponding PDB codes are indicated below each structure: 7BX7, 6WQK, 7ZIR, 6XYP, 6XYQ, 7NCA, 7NCH, 6MST, 7NCI, 7NCG, 6XYO, 6OSJ, 7NCK, 7NCJ, 7Q4B, 5KK3, 7Q4M.

Supplementary Fig. 15

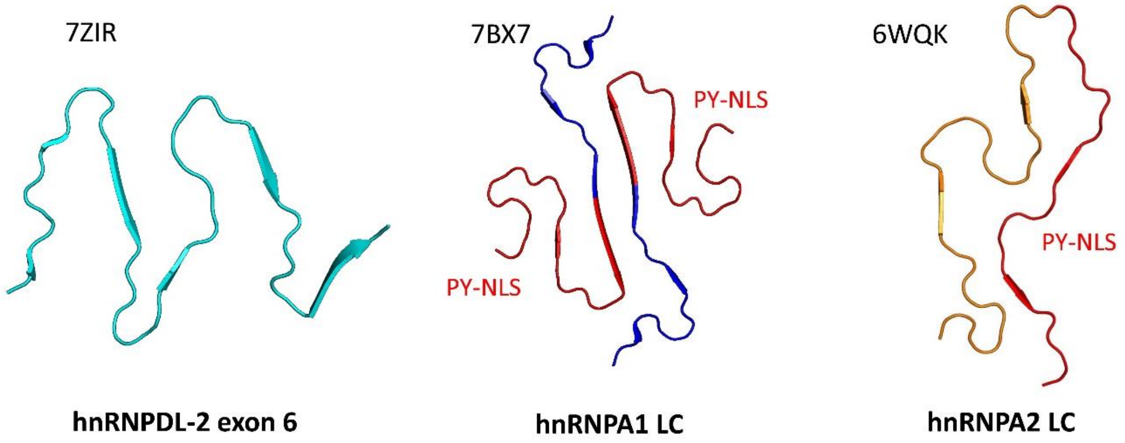

Supplementary Fig. 15. Comparison of cryo-EM structures of amyloid fibrils from RNA/DNA binding proteins. Ribbon representation of the cryo-EM amyloid core structures formed by the low complexity regions of hnRNPDL-2, hnRNPA1 and hnRNPA2. In hnRNPA1/A2, the regions corresponding to the nuclear localization sequence (PY-NLS) are colored in red. PDB codes are indicated over each structure: 7ZIR, 7BX7, 6WQK.

Supplementary Fig. 16

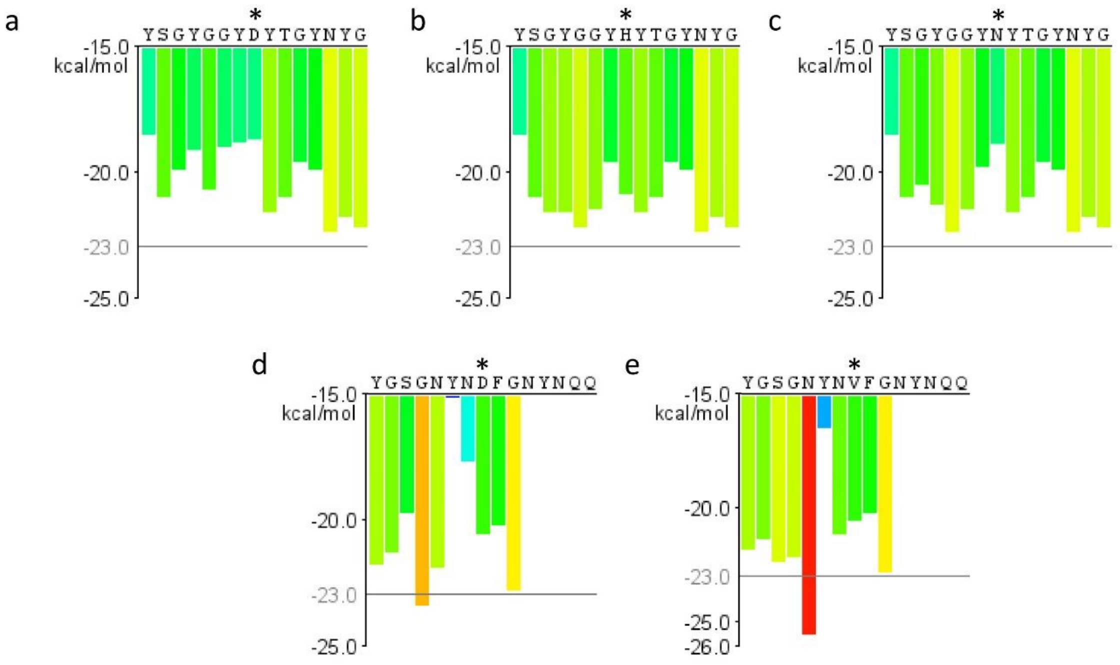

Supplementary Fig. 16. Predicted energy for fibrillation of six-residue windows of hnRNPDL-2 and hnRNPA2 disease-associated mutants. (a) hnRNPDL-2 WT, (**b**) hnRNPDL-2 D259H, (**c**) hnRNPDL-2 D259N, (**d**) hnRNPA2 WT and (**e**) hnRNPA2 D290V. Residues which are mutated in disease are indicated with an asterisk. Red bars represent hexapeptides with energy below -23kcal⁄mol, predicted to form fibrils. Data obtained from ZipperDB[4].

Supplementary Fig. 17

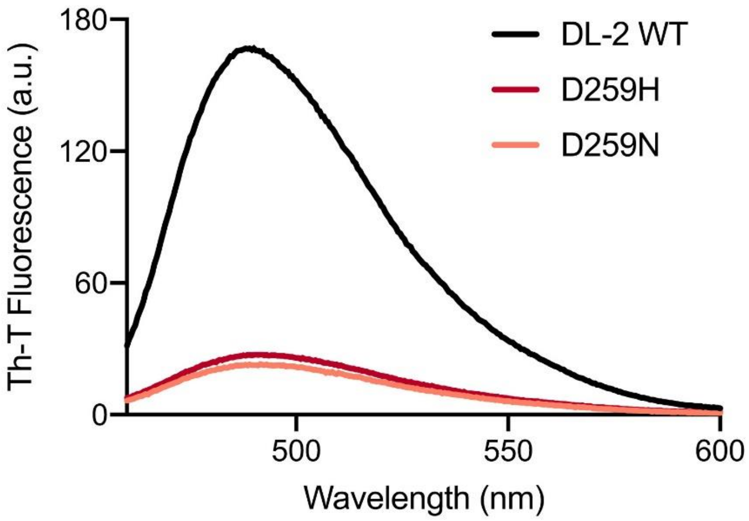

Supplementary Fig. 17. Effect of LGMD D3-associated mutations on hnRNPDL-2 fibril formation. Th-T binding to WT hnRNPDL-2 (black line) and mutant proteins D259H (red line) and D259N (pink line). All the proteins were incubated at the same conditions (37 °C, pH 7.5 and 300 mM NaCl for 48 h under constant shaking) before the Th-T binding was measured as described in the Materials and Methods section; a.u. indicates arbitrary units.

Supplementary Fig. 18

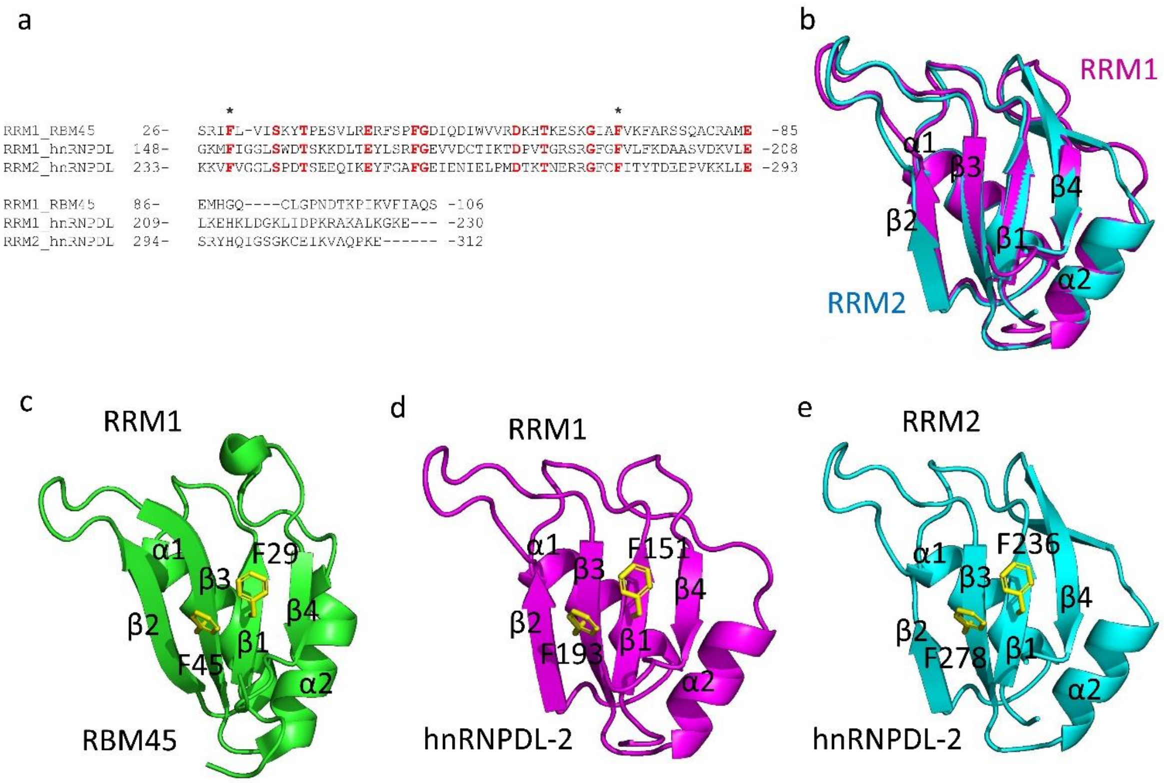

Supplementary Fig. 18. Structure of the RNA/DNA-binding domains from hnRNPDL-2. a Sequence alignment of both hnRNPDL-2 RRMs with the RRM1 from RBM45. Identical residues are highlighted in red. The positions of aromatic residues that participate in RNA/DNA binding are indicated with an asterisk. **b** Structural alignment of RRM1 and RRM2 from hnRNPDL-2. The structural models were generated with AlphaFold2[5]. **c-e** Structural comparison of the first RRM domain from RBM45 (PDB 7CSX) (**c**) and both RRM1 (**d**) and RRM2 (**e**) from hnRNPDL-2. In (**c-e**) the side chains of two of the conserved aromatic residues found in the RNA/DNA binding pocket are shown in yellow sticks.

Supplementary Fig. 19

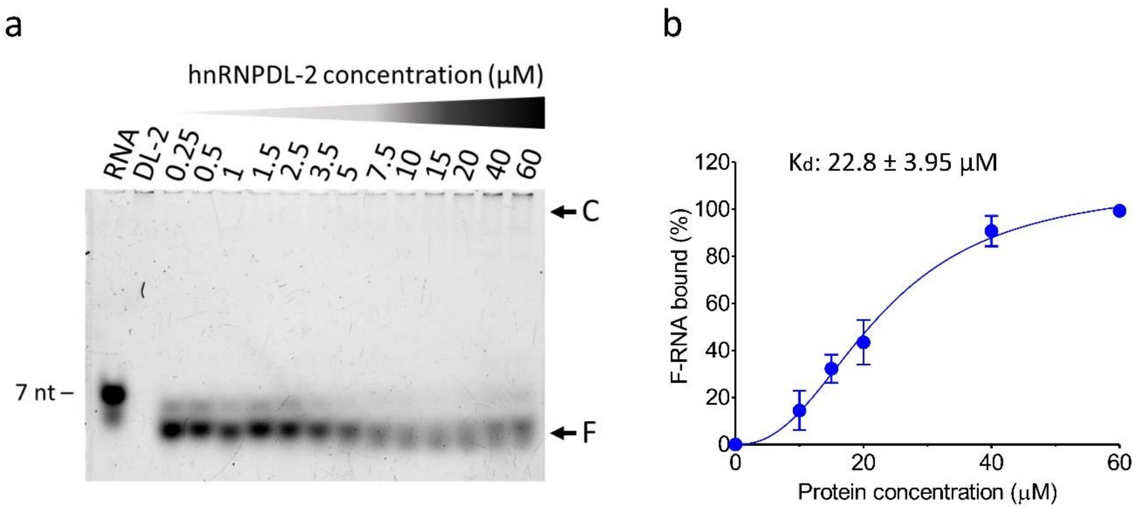

Supplementary Fig. 19. RNA (F-GACUAGC) binding to soluble hnRNPDL-2. a Electrophoretic mobility shift assay (EMSA) of soluble hnRNPDL-2 with a Fluorescein-labelled RNA (F-GACUAGC). The 7-mer RNA was incubated with increasing concentrations of the soluble form of hnRNPDL-2. The assayed protein concentrations are indicated in µM. EMSA has been performed three times with similar results. **b** Binding affinity of soluble hnRNPDL-2 to the 7-mer fluorescent RNA (F-RNA) determined by the EMSA assay. Band intensities from three independent EMSA assays were quantified and used to calculate the Kd value shown in (**b**) by assuming a one-site specific binding mechanism with Hill slope (see Materials and Methods section for equation details). In (**b**), data is shown as mean ± SEM (n = 3 independent experiments).

Supplementary Fig. 20

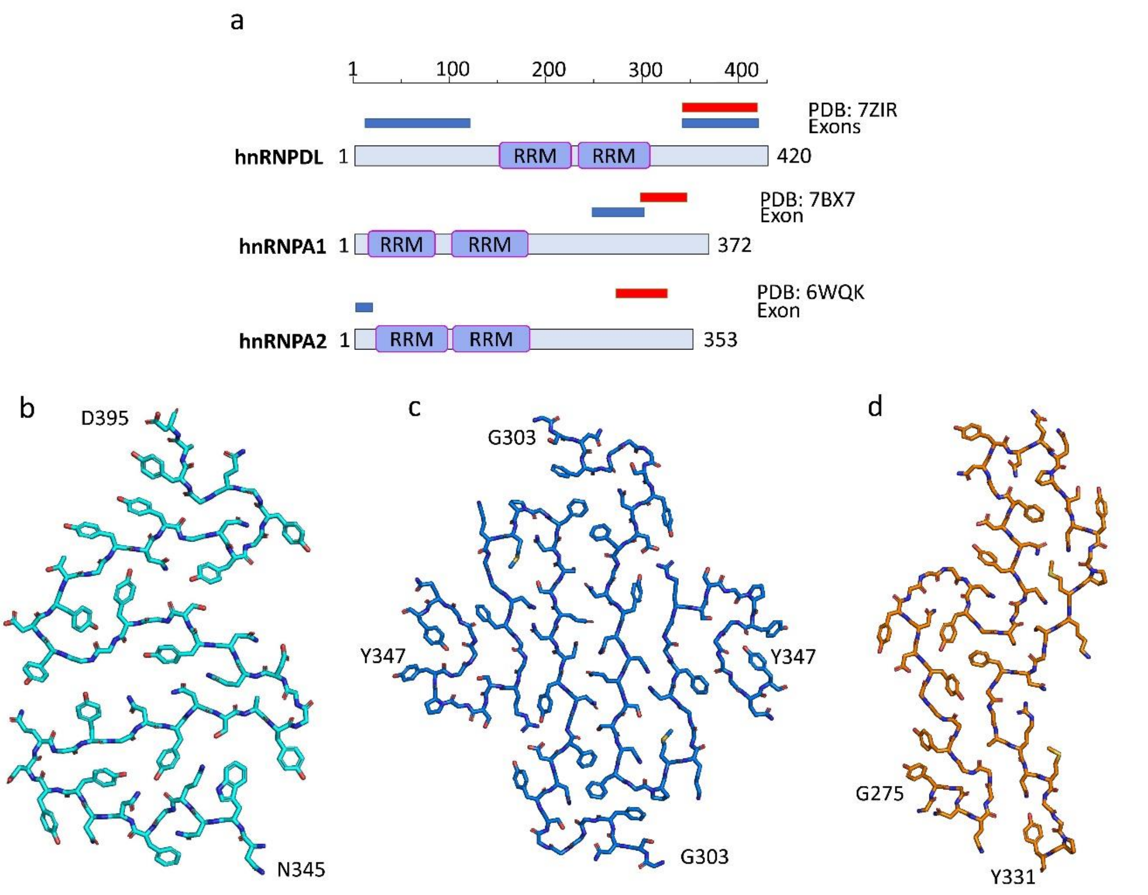

Supplementary Fig. 20. Location of amyloid cores and conserved exons in hnRNPDL, hnRNPA1 and hnRNPA2. a Primary structures of hnRNPDL, hnRNPA1 and hnRNPA2 indicating the location of the solved amyloid cores on top (red boxes). The position of exonic regions (exon/s) are also indicated over the protein structures (blue boxes). These regions correspond to the major splicing isoforms described in the Uniprot Database[6]. **b-d** Atomic models of the amyloid cores of (**b**) hnRNPDL-2 (PDB 7ZIR), (**c**) hnRNPA1 (PDB 7BX7) and (**d**) hnRNPA2 (PDB 6WQK).

SUPPLEMENTARY REFERENCES

1. Fraczkiewicz, R. & Braun, W. Exact and efficient analytical calculation of the accessible surface areas and their gradients for macromolecules. J Comp Chem 19, 319-333 (1998).
2. Krissinel, E. & Henrick, K. Inference of macromolecular assemblies from crystalline state. J Mol Biol 372, 774-97 (2007).
3. Kyte, J. & Doolittle, R.F. A simple method for displaying the hydropathic character of a protein. J Mol Biol 157, 105-32 (1982).
4. Goldschmidt, L., Teng, P.K., Riek, R. & Eisenberg, D. Identifying the amylome, proteins capable of forming amyloid-like fibrils. Proc Natl Acad Sci U S A 107, 3487-92 (2010).
5. Jumper, J. et al. Highly accurate protein structure prediction with AlphaFold. Nature 596, 583-589 (2021).
6. Bateman, A. et al. UniProt: the universal protein knowledgebase in 2021. Nucleic Acids Research 49, D480-D489 (2021).
