# Supplementary material for: Cryo-EM structure of hnRNPDL-2 fibrils, a functional amyloid associated with limb-girdle muscular dystrophy D3
Source: Nat Commun. 2023 Jan 16;14:239. doi: 10.1038/s41467-023-35854-0 (PMC9842712; doi:10.1038/s41467-023-35854-0)
Supplement: Supplementary file 3 — Reporting Summary [file 41467_2023_35854_MOESM3_ESM.pdf]

nature portfolio

Corresponding author(s): Salvador Ventura

Last updated by author(s): Dec 16, 2022

Reporting Summary

Nature Portfolio wishes to improve the reproducibility of the work that we publish. This form provides structure for consistency and transparency in reporting. For further information on Nature Portfolio policies, see our Editorial Policies and the Editorial Policy Checklist.

Statistics

For all statistical analyses, confirm that the following items are present in the figure legend, table legend, main text, or Methods section.

n/a	Confirmed	
☐	☒	The exact sample size (*n*) for each experimental group/condition, given as a discrete number and unit of measurement
☐	☒	A statement on whether measurements were taken from distinct samples or whether the same sample was measured repeatedly
☐	☒	The statistical test(s) used AND whether they are one- or two-sided<br>*Only common tests should be described solely by name; describe more complex techniques in the Methods section.*
☒	☐	A description of all covariates tested
☒	☐	A description of any assumptions or corrections, such as tests of normality and adjustment for multiple comparisons
☐	☒	A full description of the statistical parameters including central tendency (e.g. means) or other basic estimates (e.g. regression coefficient) AND variation (e.g. standard deviation) or associated estimates of uncertainty (e.g. confidence intervals)
☐	☒	For null hypothesis testing, the test statistic (e.g. *F*, *t*, *r*) with confidence intervals, effect sizes, degrees of freedom and *P* value noted<br>*Give P values as exact values whenever suitable.*
☒	☐	For Bayesian analysis, information on the choice of priors and Markov chain Monte Carlo settings
☒	☐	For hierarchical and complex designs, identification of the appropriate level for tests and full reporting of outcomes
☒	☐	Estimates of effect sizes (e.g. Cohen's *d*, Pearson's *r*), indicating how they were calculated

Our web collection on statistics for biologists contains articles on many of the points above.

Software and code

Policy information about availability of computer code

Data collection	Aggregation kinetics: Instrument- Victor3.0 Multilabel Reader (PerkinElmer) Software- PerkinElmer 2030.<br>Confocal microscopy: Instrument- Leica TCS SP5 (Leica Microsystems) Software - Bitplane Imaris 7.2.1 software.<br>Immunoblotting: Instrument- ChemiDoc™ MP (Bio-Rad Laboratoires). Software- Image Lab Touch Software (Bio-Rad Laboratoires).<br>Fluorescence spectroscopy: Instrument-Cary Eclipse Fluorescence Spectrophotometer (Varian).<br>Transmission electron microscopy: Instrument- TEM JEM-1400 (JEOL) with a CCD GATAN 794 MSC 600HP camera. Software- Gatan Digital Micrograph 1.8.<br>Cryo-electron microscopy: Instrument- 200 kV Talos Arctica TEM (Thermo Fisher Scientific) with a Falcon 3 direct electron detector (Thermo Fisher Scientific). Software- EPU 2.8 (Thermo Fisher Scientific).
Data analysis	Relion 3.1, UCSF Chimera (version 1.16), PyMOL (version 2.4.1), MOTIONCOR2, CTFFIND4, COOT, Image Lab (version 6.1.0), GraphPad Prism 5 and Image J (1.53p), Phenix (version 1.19.2) and ChimeraX (version 1.4)

For manuscripts utilizing custom algorithms or software that are central to the research but not yet described in published literature, software must be made available to editors and reviewers. We strongly encourage code deposition in a community repository (e.g. GitHub). See the Nature Portfolio guidelines for submitting code & software for further information.

Data

Policy information about availability of data

All manuscripts must include a data availability statement. This statement should provide the following information, where applicable:

- Accession codes, unique identifiers, or web links for publicly available datasets
- A description of any restrictions on data availability
- For clinical datasets or third party data, please ensure that the statement adheres to our policy

The hnRNPDL-2 amyloid fibril structure, and cryo-EM map have been deposited in the Protein Data Bank and Electron Microscopy Data Bank under the accession codes 7ZIR [http://doi.org/10.2210/pdb7ZIR/pdb] and EMDB-14738 [https://www.ebi.ac.uk/pdbe/entry/emdb/EMD-14738], respectively. Raw images and raw processing data have been deposited in Electron Microscopy Public Image Archive with accession code EMPIAR-11064 [https://www.ebi.ac.uk/empiar/EMPIAR-11064/]. Other data supporting the findings of this study are available within the article and its associated supplementary information files. Source data are provided with this paper.

Human research participants

Policy information about studies involving human research participants and Sex and Gender in Research.

Reporting on sex and gender	N/A
Population characteristics	N/A
Recruitment	N/A
Ethics oversight	N/A

Note that full information on the approval of the study protocol must also be provided in the manuscript.

Field-specific reporting

Please select the one below that is the best fit for your research. If you are not sure, read the appropriate sections before making your selection.

☒ Life sciences ☐ Behavioural & social sciences ☐ Ecological, evolutionary & environmental sciences

For a reference copy of the document with all sections, see nature.com/documents/nr-reporting-summary-flat.pdf

Life sciences study design

All studies must disclose on these points even when the disclosure is negative.

Sample size	No statistical methods were used to predetermine sample sizes. Sample sizes were determined based on previous publications on similar experiments. For cryoEM experiments (Sun Y, et al. Nature Communications, 2020, doi: 10.1038/s41467-020-20227-8; Lu J, et al. Nature Communications, 2020. doi: 10.1038/s41467-020-17905-y). For aggregation kinetics analysis (Pujols et. al. Proc Natl Acad Sci U S A, 2018, doi: 10.1073/pnas.1804198115), and for Cell-based assays (Batlle et. al. Science Reports, 2020, doi: 10.1016/j.celrep.2019.12.080). The range of concentrations used to determine the cytotoxicity of the hnRNPDL-2 fibers was based on other similar papers (Diaz-Caballero M, et al. 2020, Biomacromolecules, doi: 10.1021/acs.biomac.0c00271).
Data exclusions	In single-particle cryo-EM data processing so called 'bad micrographs' have been excluded due to their obvious poor quality. During processing, particles that does not contribute to best 3D classes have been also excluded.
Replication	All the experiments were repeated at least twice or thrice obtaining similar results, if not stated otherwise in the methods section.
Randomization	This does not apply to the present study since only one homogeneous state was targeted.
Blinding	Blinding is not relevant to our experiments because structural data collection and analysis is not biased by investigators. For the rest of experiments, blinding is not possible since the investigators who analyzed the data also performed the experiments.

Behavioural & social sciences study design

All studies must disclose on these points even when the disclosure is negative.

Study description	*Briefly describe the study type including whether data are quantitative, qualitative, or mixed-methods (e.g. qualitative cross-sectional, quantitative experimental, mixed-methods case study).*
Research sample	*State the research sample (e.g. Harvard university undergraduates, villagers in rural India) and provide relevant demographic information (e.g. age, sex) and indicate whether the sample is representative. Provide a rationale for the study sample chosen. For studies involving existing datasets, please describe the dataset and source.*
Sampling strategy	*Describe the sampling procedure (e.g. random, snowball, stratified, convenience). Describe the statistical methods that were used to predetermine sample size OR if no sample-size calculation was performed, describe how sample sizes were chosen and provide a rationale for why these sample sizes are sufficient. For qualitative data, please indicate whether data saturation was considered, and what criteria were used to decide that no further sampling was needed.*
Data collection	*Provide details about the data collection procedure, including the instruments or devices used to record the data (e.g. pen and paper, computer, eye tracker, video or audio equipment) whether anyone was present besides the participant(s) and the researcher, and whether the researcher was blind to experimental condition and/or the study hypothesis during data collection.*
Timing	*Indicate the start and stop dates of data collection. If there is a gap between collection periods, state the dates for each sample cohort.*
Data exclusions	*If no data were excluded from the analyses, state so OR if data were excluded, provide the exact number of exclusions and the rationale behind them, indicating whether exclusion criteria were pre-established.*
Non-participation	*State how many participants dropped out/declined participation and the reason(s) given OR provide response rate OR state that no participants dropped out/declined participation.*
Randomization	*If participants were not allocated into experimental groups, state so OR describe how participants were allocated to groups, and if allocation was not random, describe how covariates were controlled.*

Ecological, evolutionary & environmental sciences study design

All studies must disclose on these points even when the disclosure is negative.

Study description	*Briefly describe the study. For quantitative data include treatment factors and interactions, design structure (e.g. factorial, nested, hierarchical), nature and number of experimental units and replicates.*
Research sample	*Describe the research sample (e.g. a group of tagged Passer domesticus, all Stenocereus thurberi within Organ Pipe Cactus National Monument), and provide a rationale for the sample choice. When relevant, describe the organism taxa, source, sex, age range and any manipulations. State what population the sample is meant to represent when applicable. For studies involving existing datasets, describe the data and its source.*
Sampling strategy	*Note the sampling procedure. Describe the statistical methods that were used to predetermine sample size OR if no sample-size calculation was performed, describe how sample sizes were chosen and provide a rationale for why these sample sizes are sufficient.*
Data collection	*Describe the data collection procedure, including who recorded the data and how.*
Timing and spatial scale	*Indicate the start and stop dates of data collection, noting the frequency and periodicity of sampling and providing a rationale for these choices. If there is a gap between collection periods, state the dates for each sample cohort. Specify the spatial scale from which the data are taken*
Data exclusions	*If no data were excluded from the analyses, state so OR if data were excluded, describe the exclusions and the rationale behind them, indicating whether exclusion criteria were pre-established.*
Reproducibility	*Describe the measures taken to verify the reproducibility of experimental findings. For each experiment, note whether any attempts to repeat the experiment failed OR state that all attempts to repeat the experiment were successful.*
Randomization	*Describe how samples/organisms/participants were allocated into groups. If allocation was not random, describe how covariates were controlled. If this is not relevant to your study, explain why.*
Blinding	*Describe the extent of blinding used during data acquisition and analysis. If blinding was not possible, describe why OR explain why blinding was not relevant to your study.*

Did the study involve field work? ☐ Yes ☐ No

Field work, collection and transport

Field conditions	*Describe the study conditions for field work, providing relevant parameters (e.g. temperature, rainfall).*
Location	*State the location of the sampling or experiment, providing relevant parameters (e.g. latitude and longitude, elevation, water depth).*
Access & import/export	*Describe the efforts you have made to access habitats and to collect and import/export your samples in a responsible manner and in compliance with local, national and international laws, noting any permits that were obtained (give the name of the issuing authority, the date of issue, and any identifying information).*
Disturbance	*Describe any disturbance caused by the study and how it was minimized.*

Reporting for specific materials, systems and methods

We require information from authors about some types of materials, experimental systems and methods used in many studies. Here, indicate whether each material, system or method listed is relevant to your study. If you are not sure if a list item applies to your research, read the appropriate section before selecting a response.

Materials & experimental systems

n/a	Involved in the study	
☐	☒	Antibodies
☐	☒	Eukaryotic cell lines
☒	☐	Palaeontology and archaeology
☒	☐	Animals and other organisms
☒	☐	Clinical data
☒	☐	Dual use research of concern

Methods

n/a	Involved in the study	
☒	☐	ChIP-seq
☒	☐	Flow cytometry
☒	☐	MRI-based neuroimaging

Antibodies

Antibodies used

Antibody list (suppliers, catalogue numbers and dilutions): anti-hnRNPDL antibody, catalogue number HPA056820 from Sigma-Aldrich (1:500 dilution); anti-6xHis, catalogue number MA1-21315 from Thermo-Fisher (dilution 1:10); anti-vinculin monoclonal antibody VLN01, catalogue number MA5-11690 from Invitrogen (dilution 1:5000); goat anti-Mouse IgG (H+L) Secondary Antibody HRP, catalogue number 31430 from Thermo-Fisher Scientific (dilution 1:2000); goat anti-Rabbit IgG (H+L) Secondary Antibody HRP, catalogue number 31460 from Thermo-Fisher Scientific (dilution 1:2000); goat anti-mouse 10-nm colloidal gold linked secondary antibody, catalog number A-31561 from Thermo-Fisher Scientific (dilution 1:100).

Validation

All the antibodies used in this study were commercial antibodies and were only used for applications with validation procedures described on the corresponding sites of the manufacturer's. The links to the manufacturer's website and the corresponding validations of the primary antibodies are are provided below:

anti-hnRNPDL antibody:
https://www.sigmaaldrich.com/ES/es/product/sigma/hpa056820

anti-6xHis antibody:
https://www.thermofisher.com/antibody/product/6x-His-Tag-Antibody-clone-HIS-H8-Monoclonal/MA1-21315

anti-Vinculin monoclonal antibody:
https://www.thermofisher.com/antibody/product/Vinculin-Antibody-clone-VLN01-Monoclonal/MA5-11690

Eukaryotic cell lines

Policy information about cell lines and Sex and Gender in Research

Cell line source(s)	HeLa (ATCC CCL-22; RRID: CVCL_0030) cells were acquired from A.T.C.C. (VA, USA).<br>SH-SY5Y (ATCC CRL-2266) human neuroblastoma were acquired from A.T.C.C. (VA, USA).
Authentication	HeLa cells have been authenticated within the last 3 years by STR analysis.
Mycoplasma contamination	Cells were regularly tested for the presence of mycoplasma using commercial PCR-based detection kits. All the cell lines used in the present study are mycoplasma negative.

Commonly misidentified lines<br>(See ICLAC register)	HeLa cells
